# Contrasting Satellitomes in New World and African Trogons (Aves, Trogoniformes)

**DOI:** 10.3390/genes16111301

**Published:** 2025-11-01

**Authors:** Luciano Cesar Pozzobon, Jhon Alex Dziechciarz Vidal, Felipe Lagreca Bitencour, Analía Del Valle Garnero, Ricardo José Gunski, Hélio Gomes da Silva Filho, Fabio Porto-Foresti, Ricardo Utsunomia, Marcelo de Bello Cioffi, Thales Renato Ochotorena de Freitas, Rafael Kretschmer

**Affiliations:** 1 Laboratório de Citogenética e Evolução, Departamento de Genética, Instituto de Biociências, Universidade Federal do Rio Grande do Sul, Porto Alegre 91509-900, Brazil; lcpozzobon48@gmail.com (L.C.P.); thales.freitas@ufrgs.br (T.R.O.d.F.); 2 Laboratório de Citogenética Evolutiva, Departamento de Genética e Evolução, Universidade Federal de São Carlos, São Carlos 13565-905, Brazil; jhonalex279@gmail.com (J.A.D.V.); mbcioffi@ufscar.br (M.d.B.C.); 3 Laboratório de Diversidade Genética Animal, Universidade Federal do Pampa, São Gabriel 97300-000, Brazil; analiagarnero@unipampa.edu.br (A.D.V.G.); ricardogunski@unipampa.edu.br (R.J.G.); 4 Faculdade de Ciências, Universidade Estadual Paulista, Bauru 13506-900, Brazil; hg.silva@unesp.br (H.G.d.S.F.); fp.foresti@unesp.br (F.P.-F.); ricardo.utsunomia@unesp.br (R.U.)

**Keywords:** repetitive DNA, satellite DNA, genome evolution, sex chromosome differentiation, speciation, birds

## Abstract

Background/Objectives: Satellite DNAs (satDNAs) are tandemly repeated sequences that play essential roles in chromosome structure, genome organization, and evolution. Despite their importance, the satellitome (the complete collection of satDNAs) of most avian lineages remains unexplored. We sought to describe the repeatome of three trogonid species, *Trogon surrucura*, *T*. *melanurus*, and *Apaloderma vittatum* with a focus on the satellitome to evaluate the general features of this lineage. Methods: Herein, we provide the first comparative characterization of the repeatome, with a particular focus on the comparative characterization of satDNAs in three trogonid species: *T. surrucura*, *T*. *melanurus*, and *A. vittatum*. Using a combination of bioinformatic pipelines and cytogenetic approaches. Results: We identified 16 satDNA families in *T*. *surrucura*, 15 in *T*. *melanurus*, and only 3 in *A*. *vittatum*. Sequence comparisons revealed that five families are shared between the two *Trogon* species, consistent with the library hypothesis, whereas no satDNAs were shared with *A*. *vittatum*. While both *Trogon* species exhibited a predominance of GC-rich repeats, *A*. *vittatum* represents the first bird described with a satellitome dominated by AT-rich satDNAs. In situ mapping in *T*. *surrucura* revealed chromosome-specific satDNAs restricted to pairs 1 and 2 and a Z-specific repeat that was strongly accumulated on its long arms, an atypical feature among birds. Conversely, the W chromosome showed a surprisingly low number of satDNAs, limited to centromeric signals. Conclusions: Our results reveal highly divergent satellitome landscapes among trogonids, characterized by lineage-specific differences in repeat composition, abundance, and chromosomal distribution. These findings support the view that satDNAs are dynamic genomic elements, whose amplification, loss, and chromosomal redistribution can influence genome architecture and play a role in avian speciation.

## 1. Introduction

Repetitive DNA has become a valuable resource for understanding genome evolution, demonstrating a fundamental role in genome structure [1]. The most extensively studied class of repetitive DNA is transposable elements (TEs), owing to their high interspecific diversity, distinct patterns of genomic distribution, and multiple functional roles, including gene regulation, shaping 3D chromatin architecture, and influencing genome size [2]. In addition, recent studies have highlighted another class of repetitive DNA with major relevance to genome evolution: satellite DNAs (satDNAs). In contrast to TEs, which are dispersed throughout the genome, satDNAs consist of tandemly repeated sequences organized in a head-to-tail pattern, forming large arrays extending over several megabases. These sequences are widely distributed across plant and animal genomes, generally located in the centromeric, pericentromeric, and subtelomeric regions of chromosomes [3,4], and may account for a great abundance in eukaryotic genomes [5,6].

The evolutionary dynamics of satDNAs are shaped by several non-exclusive processes. The library hypothesis suggests that closely related species share a common repertoire of satDNA families, although the abundance of each family may vary across lineages [3,7,8,9,10]. The concerted evolution model predicts that satDNA monomers evolve in a coordinated manner within a genome due to mechanisms such as unequal crossing-over, gene conversion, and intrachromosomal rolling circle amplification [4,5,11,12,13,14,15,16]. These dynamics generate heterogeneous satellitome landscapes, where some families remain conserved across taxa while others are species-specific [4,8,9,13]. Importantly, satDNAs have been implicated in speciation, as rapid divergence in repeat composition can reduce chromosomal homology, promote structural rearrangements, and contribute to reproductive isolation [17,18,19,20,21].

In birds, satDNAs are most frequently located in centromeric regions and microchromosomes, although lineage-specific exceptions are being increasingly reported [22,23,24,25,26,27]. Despite their small size, microchromosomes are gene-rich compared to macrochromosomes [28] and may provide unique environments for the accumulation or retention of repeats. In avian sex chromosomes (ZZ/ZW system), the W chromosome predisposes it to accumulating repetitive DNA due to its haploid state [29]. In most bird species, the Z chromosome typically exhibits satDNAs confined to the centromeric region [24,25], whereas the W chromosome often contains W-specific repeats distributed across both centromeric and interstitial regions [22,23,27]. For example, in *Gallus gallus*, the W chromosome alone accounts for more than 30% of the satellitome [28].

Trogonidae (Aves, Trogoniformes) comprises seven genera and approximately 46 species of pantropical forest birds in Africa, Asia, and the Americas [30]. They are characterized by marked sexual dimorphism, with males exhibiting vivid plumage and elongated tail feathers. Despite diverging from their sister groups in the Paleocene (~35 Mya), trogons underwent a more recent radiation at the Oligocene–Miocene boundary (~23 Mya) [31]. Cytogenetic data for this group are scarce, with only two species being previously karyotyped: *Harpactes erythrocephalus* (2n = 76) [32] and *T. surrucura* (2n = 82) [33]. As is typical for birds, these karyotypes are composed of a few morphologically distinct macrochromosomes and numerous, indistinguishable microchromosomes [34,35,36]. Although *T. surrucura* falls within the typical avian karyotypic range (2n = 78–82) [37], it presents several unusual features, including a W chromosome similar in size to the Z [33], accumulation of the retroelement AviRTE on both sex chromosomes [38], multiple rDNA sites [33], and evidence of chromosomal fusions, fissions, and inversions [33,39]. Together, these characteristics highlight this lineage’s potential for atypical repeat dynamics.

In this study, we present the first comparative characterization of the repeatome, with a particular focus on the satellitome, in three Trogonidae species (*T. surrucura, T. melanurus,* and *A. vittatum*). Special attention is devoted to the sex chromosomes of *T. surrucura*, enabling us to evaluate whether the satellitome of Trogonidae follows general avian patterns or exhibits lineage-specific innovations. Specifically, we sought to (I) describe the sequence composition, diversity, and abundance of satDNA families, (II) assess the extent of repeat sharing across species, and (III) investigate their chromosomal distribution using both in silico and in situ approaches, and (IV) finally, we compared the differences within Trogonidae and among birds with available literature.

## 2. Materials and Methods

### 2.1. Chromosome Sampling

Metaphase chromosome spreads were obtained from fibroblast cell culture established from the feather pulp of one female individual of *T. surrucura*, collected on Caçapava do Sul (RS, Brazil), following [40]. Cell culture was treated with colchicine 0.05% for 1 h, suspended and incubated with hypotonic solution (0.075 mol/L KCl) for 8 min at 37 °C, finally washed and fixed with Carnoy I (3:1 methanol and acetic acid). The experiments were approved by Sistema de Autorização e Informação em Biodiversidade (SISBIO 44173-3 and 61047-3) and followed protocols approved by the ethics committee from Universidade Federal do Pampa (no. 018/2014 and 019/2020).

### 2.2. Genomic DNA Sequencing and Bioinformatic Analysis

Genomic DNA (gDNA) was extracted from the blood of a single female individual of *T. surrucura* using the PureLink™ Genomic DNA Mini Kit (Invitrogen, Carlsbad, CA, USA). After that, gDNA was sequenced on the BGISEQ-500 platform at BGI (BGI Shenzhen Corporation, Shenzhen, China) (PE-150), yielding around 3 Gb, representing ~3× coverage. The raw reads were submitted to the Sequence Read Archive (SRA-NCBI) under the accession number SRR35639635. To enable a comparative analysis of satDNA within Trogonidae, we retrieved two additional short-read datasets from the public SRA-NCBI repository: *T. melanurus* (SRR9947003) and *A. vittatum* (SRR952778). The complete set of satDNA sequences characterized in this study for *T. surrucura* (PX241785-PX241800), *T. melanurus* (PX311080-PX311094), and *A. vittatum* (under submission on NCBI) has been submitted to GenBank.

To evaluate the complete composition of repetitive DNA, the assembled genomes of *T. surrucura, T. melanurus*, and *A. vittatum* available in NCBI, under the accession numbers GCA_020746105.1, GCA_013399275.1, and GCA_000703405.1, respectively, were analyzed. Repetitive elements were identified through de novo annotation using RepeatModeler [41]. The results were then complemented with the satDNAs identified in this study to improve the accuracy of the annotations. The overall composition of repetitive DNA was calculated using RepeatMasker [42]. The Kimura-2-parameter (K2P) divergence values for each transposable element were calculated using the calcDivergenceFromAlign.pl script in the RepeatMasker package [42].

The satDNAs were characterized following the pipeline described by [3] using the Tandem Repeat Analysis (TAREAN) tool [43]. Raw reads were first quality-filtered using Trimmomatic 0.40 [44]. The trimmed libraries were subsampled to 2 × 500,000 randomly selected reads using SeqTK (https://github.com/lh3/seqtk, accessed on 15 November 2024) and analyzed with TAREAN. Putative satDNAs identified in this step were then used to filter the trimmed libraries with DeconSeq [45]. New filtered libraries were again subsampled (2 × 500,000 reads) and submitted to TAREAN. This iterative process was repeated until no additional satDNAs were detected.

Because multigene family sequences are highly repetitive, putative satDNAs were aligned against known multigene families in GENEIOUS 6.1.8 (Biomatters, Auckland, New Zealand), and any sequences showing similarity to known multigene families (>80%) were removed. To eliminate redundancy, the set of satDNAs was aligned against itself using the cross_match search from RepeatMasker [42], and duplicate sequences were discarded.

SatDNA abundances were estimated using RepeatMasker (https://github.com/fjruizruano/satminer/blob/master/repeat_masker_run_big.py, accessed on 15 November 2024) by aligning 10,000,000 (2 × 5,000,000 reads) randomly selected trimmed reads against the final satDNA library. Sequence divergence for each satDNA family was calculated with the calcDivergenceFromAlign.pl script [3] using the Kimura 2-parameter (K2P) correction. Final satDNA families were named according to the convention of [3], ranking sequences by decreasing genomic abundance and including the first letter of the genus and the first two letters of the species epithet.

To investigate the interspecific sharing of satDNAs among *T. surrucura*, *T. melanurus*, and *A. vittatum*, we conducted comparative analyses of their satellitomes. Consensus sequences from all species-specific catalogs were concatenated and converted into dimer format using custom scripts. Sequence similarity was first assessed with RepeatMasker, and homologous clusters were refined through manual curation using the MUSCLE algorithm [46]. Satellite DNAs exhibiting ≥ 80% sequence similarity were classified as variants of the same satDNA family. Local alignments were applied when comparing sequences of different lengths, whereas global alignments were used for sequences of identical size.

To investigate the in silico distribution of satellite DNAs in Trogonidae, we analyzed the first ten autosomal chromosomes along with the sex chromosomes of *T. surrucura.* The analyses were performed using the CHRISMAPP pipeline (CHRromosome In Silico MAPPing; https://github.com/LoriteLab/CHRISMAPP, accessed on 15 November 2024), following the approach described by Rico-Porras et al. [47]. For this purpose, we employed the chromosome-level genome assembly available in NCBI (accession GCA_020746105.1).

### 2.3. Probes and In Situ Mapping of TsuSatDNAs

Primers were designed for 9 out of 16 TsuSatDNAs, while the remaining 7 were labeled with 5′ biotin during synthesis by Exxtend (Paulínia, Brazil) (Table S1). The TsuSatDNAs were amplified by Polymerase Chain Reaction (PCR), which included an initial denaturation of 95 °C for 3 min, 35 cycles of denaturation at 95 °C for 45 s, annealing at 51–58 °C for 35 s, and extension at 72 °C for 45 s, and a final extension of 72 °C for 5 min (Table S2). The integrity of the PCR products was analyzed on 2% agarose gel. The probes were further biotin-labeled with BioNick™ DNA Labeling System (Invitrogen, Carlsbad, CA, USA). Mapping of TsuSatDNAs was performed following the Fluorescence in situ Hybridization (FISH) protocol described by [48]. The probes were detected with Streptavidin-Cy3 dye, and the chromosomes were counterstained with 4′,6-diamidino-2-phenylindole (DAPI) solution. Given that the W chromosome contains a highly distinct C-positive heterochromatic block, which allows it to be readily distinguished from other chromosomes, the presence of TsuSatDNAs on the W chromosome was confirmed by performing C-banding after FISH experiments, following the protocol of [49]. At least 15 metaphases were examined on the fluorescence microscope Zeiss Axiophot (ZEISS Inc., Carl Zeiss, Heidelberg, Germany), equipped with the camera AxioCam MRm and ZEN 2 (blue edition) (ZEISS Inc.). The images were subsequently edited with CorelDraw v.23.1.0.389.

## 3. Results

### 3.1. Composition of Repetitive DNA in Trogonidae

**Figure 1:**
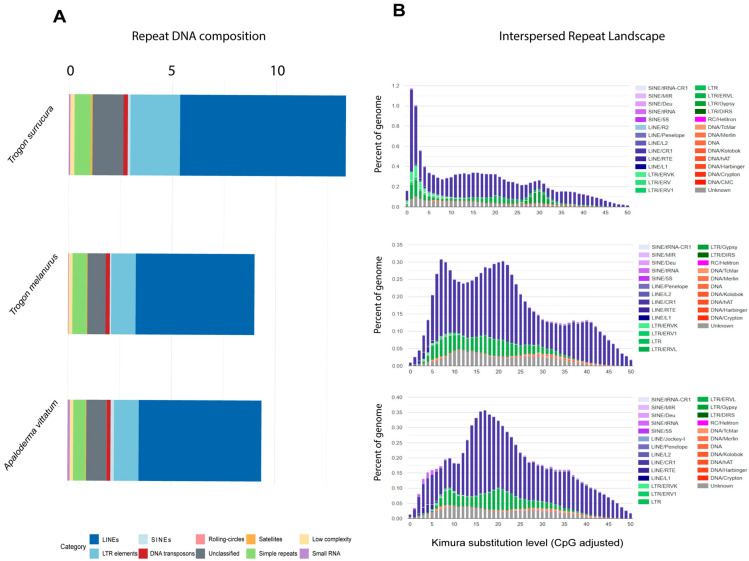
Repetitive landscape of Trogonidae species: percentage of repetitive DNA in the genome of each species (**A**) and repeat landscape using Kimura substitution (**B**).

Among the Trogoniformes, genome assemblies currently available in NCBI include *T. surrucura* at the chromosome level, whereas *T. melanurus* and *A. vittatum* are represented only at the scaffold level. The overall proportion of repetitive DNA was highest in *T. surrucura* (13.29%), followed by *A. vittatum* (9.24%) and *T. melanurus* (8.98%). Despite these differences in total abundance, the repeat composition was similar across species, with LINEs (retroelements) being the most abundant, followed by LTR elements and unclassified repeats. Among LINEs, CR1 elements were predominant, accounting for 7.88%, 5.66%, and 5.81% of the genome in *T. surrucura*, *T. melanurus*, and *A. vittatum*, respectively (Figure 1A).

Kimura-based repeat landscape analyses revealed distinct evolutionary dynamics among the three species. *T. surrucura* showed a large number of repeats linked to very recent amplification (<5% Kimura divergence). In contrast, *T. melanurus* exhibited two prominent amplification peaks at approximately 5% and 23% divergence, while *A. vittatum* lacked evidence of recent expansion, with its repeat content dominated by more ancient elements (Figure 1B).

### 3.2. Satellite DNAs of Trogonidae

The analysis of satellite DNA (satDNA) in *T. surrucura*, *T. melanurus*, and *A. vittatum* revealed notable similarities between *T. surrucura* and *T. melanurus.* These two species contained 16 and 15 satDNA families, representing approximately 7.5% and 4.8% of their respective genomes (Table 1 and Table 2). In contrast, *A. vittatum* exhibited a markedly lower diversity, with only three satDNA families, accounting for roughly 1.8% of its genome (Table 3). Unlike the *Trogon* species, whose satDNA is predominantly GC-rich, *A. vittatum* displayed a high A + T content, representing a distinctive feature within Trogonidae (Table 1, Table 2 and Table 3). The satDNA landscape of *T. surrucura* is characterized by a recent amplification (0–5% Kimura). In *T. melanurus* and *A. vittatum*, the landscapes showed a broader distribution, with a major peak at ~5% and ~7% divergence, respectively, suggesting a slightly older expansion (Figure S1).

The three species exhibited comparable distributions of satDNA family sizes. In *T. surrucura*, *T. melanurus*, and *A. vittatum*, 9, 5, and 1 monomers were composed of short sequences (<100 bp), whereas 7, 8, and 2 monomers consisted of long sequences (>100 bp), respectively. The most pronounced differences in satDNA length were observed in *T. melanurus*, where TmeSat03-2458 and TmeSat06-2022 exhibited exceptionally large sizes (Table 1, Table 2 and Table 3).

Comparative analysis revealed that *T. surrucura* and *T. melanurus* share five families, each exhibiting over 80% sequence similarity, suggesting conservation since their divergence (Table 4). Additionally, four superfamilies were identified, displaying sequence similarities ranging from 69% to 77%. In contrast, *A. vittatum* shared no satDNA families with either *T. surrucura* or *T. melanurus*, indicating a distinct and unique satDNA repertoire.

### 3.3. In Silico Mapping of TsuSatDNAs and TmeSatDNAs

**Figure 2:**
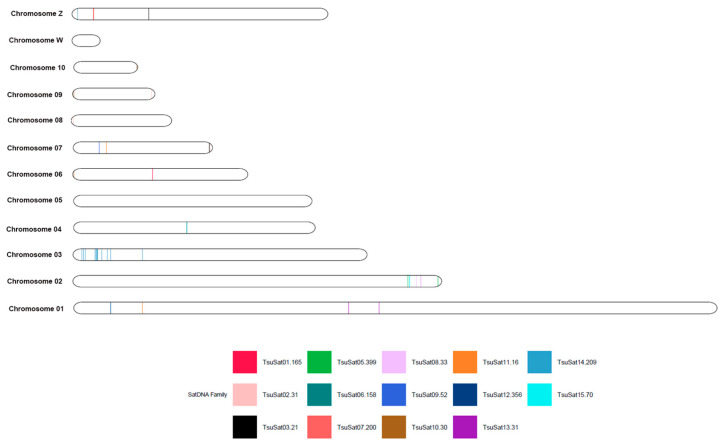
In silico mapping of TsuSatDNAs on *T. surrucura*.

**Figure 3:**
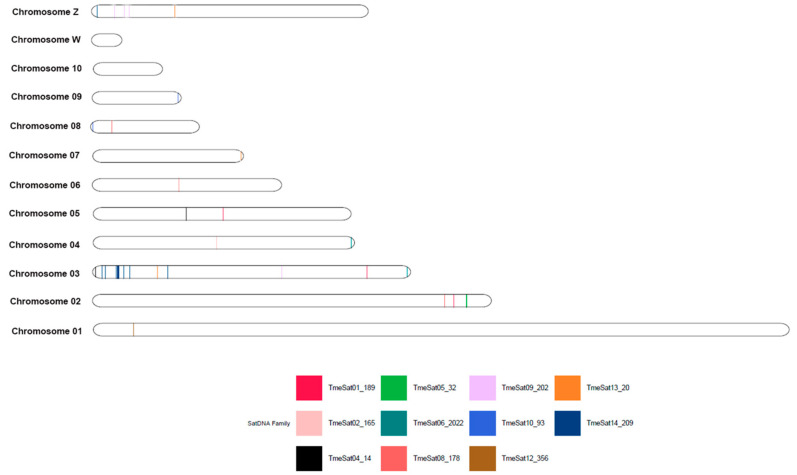
In silico mapping of *T. melanurus* satDNA families.

The in silico mapping of TsuSatDNAs (Figure 2) and TmeSatDNAs (Figure 3) in the first ten and ZW chromosomes showed the presence of the satDNAs on 8 macrochromosomes of autosomes and on Z chromosomes of both species. Additionally, there is no presence of satDNAs on chromosome W of both species, as well as on chromosome 5 in *T. surrucura* and chromosome 10 in *T. melanurus*. Furthermore, the satellites TsuSat04-31 and TsuSat16-105 of *T. surrucura*, and TmeSat03-2458, TmeSat07-219, TmeSat11-502, and TmeSat15-37 of *T. melanurus* were not identified on the macrochromosomes.

### 3.4. In Situ Mapping of TsuSatDNAs

**Figure 4:**
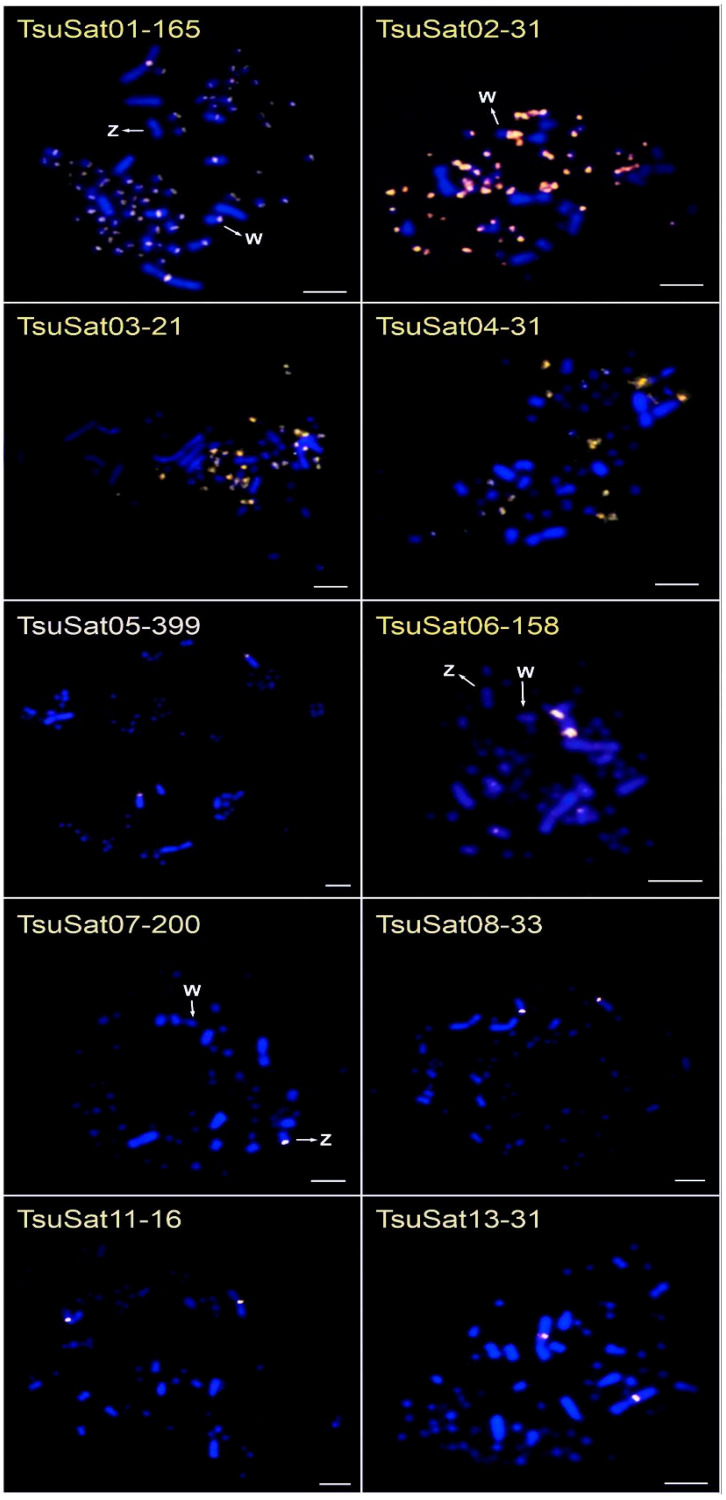
Satellite DNA of *T. surrucura* hybridized to female metaphase chromosomes. Blue: chromosomes counterstained with DAPI; Yellow and white colors: TsuSatDNA probes. Bar = 5 µm.

Of the 16 satDNA families characterized, 13 were successfully mapped onto the chromosomes of a female *T. surrucura* (Figure 4; Table 5). The most abundant families exhibited hybridization signals across multiple chromosomes. Notably, TsuSat01-165 was distributed across nearly all chromosomes, except for the W chromosome and pair 2. Similarly, TsuSat02-31 was detected on most chromosomes but was absent from the Z chromosome and the first seven macrochromosome pairs. In contrast, TsuSat03-21 and TsuSat04-31 showed exclusive localization to microchromosomes (Figure 4; Table 5).

The satDNA families TsuSat05-399 and TsuSat08-33 hybridized to the centromeric region of pair 2. A strong signal for TsuSat06-158 was observed on pair 3, with weaker signals on other chromosomes, but was completely absent on pair 2 and the Z chromosome. TsuSat07-200 was localized to the q arm of the Z chromosome, while TsuSat11-16 and TsuSat13-31 hybridized exclusively to pair 1 (Figure 4; Table 5).

Finally, three satDNA families (TsuSat10-30, TsuSat14-209, and TsuSat16-105) exhibited no detectable hybridization signals, suggesting that these sequences may be dispersed throughout the genome or present in clusters too small to be detected by FISH.

## 4. Discussion

In this study, we characterized the repetitive genome content of three Trogonidae species, focusing on the satellitome: *T. surrucura* (16 satDNAs), *T. melanurus* (15 satDNAs), and *A. vittatum* (3 satDNAs). We also performed their in silico mapping on the first 10 chromosomes and sex chromosomes of *T. surrucura* and *T. melanurus*, complemented by in situ mapping in *T. surrucura* chromosomes. Most abundant satDNAs were located in centromeric regions across multiple chromosomes, with three satDNAs specific to chromosome 2, while TsuSat07-200 was uniquely mapped on the long arms of the Z chromosome. Additionally, our cytogenetic analyses confirmed the diploid number of *T. surrucura* (2n = 82), as previously described by [33].

### 4.1. Repetitive DNAs in Trogonidae

Vertebrate genomes are generally large; however, birds are known to have relatively small genomes, ranging from 0.9 to 2.2 pg and averaging about 1.4 pg [50,51]. The primary explanation for this difference in genome size between birds and most other vertebrates is their low proportion of repetitive DNAs, as avian genomes contain comparatively few repetitive elements [34]. Transposable element (TE) content in birds is typically around 7–10%, with only a few species harboring higher amounts [50]. Among TEs, long interspersed nuclear elements (LINEs) are the most abundant in Trogonidae species, as also observed in other birds [52,53]. LINEs underwent major expansions in two principal vertebrate clades—mammals and birds [50,54]. In birds, the CR1 family is the key driver of LINE proliferation and is considered crucial for avian genome evolution, as it is conserved across the avian phylogeny while displaying lineage-specific expansion rates [55]. Consistently, Trogoniformes exhibit a high proportion of CR1 retroelements, representing 7.88%, 5.66%, and 5.81% of the genomes of *T. surrucura*, *T. melanurus*, and *A. vittatum*, respectively, highlighting the important role of CR1 in genome architecture and evolution within the Trogonidae.

### 4.2. General Features of satDNAs in Trogonidae

The content of satDNA on bird genomes can vary significantly. For instance, *Corvus splendens* contains up to 28 distinct satDNA families [9]. In contrast, *Sula leucogaster,* despite comprising only five satDNA families, exhibits one of the highest genomic proportions of satDNA reported to date, accounting for 17.99% of its genome [27]. Interestingly, while *T. surrucura* and *T. melanurus* differed by only one satDNA family, their overall satDNA content varied greatly: 7.55% in *T. surrucura* (Table 1) versus 4.89% in *T. melanurus* (Table 2). In contrast, *A. vittatum* reported the lowest number of satDNA families in birds to date and 1.85% satDNA content (Table 3), while *Vanellus chilensis*, with seven families, presented only 0.92% [22]. These differences suggest that satDNA amplification dynamics contribute to genomic differentiation and may potentially lead to reproductive isolation among related species.

Despite their divergence time of 15.4 million years (My) [56], *T. surrucura* and *T. melanurus* shared five satDNA families, with sequence similarities of 84–98% (Table 4). Three of these variants had identical monomer size, and two differed by only 1–2 bp. In contrast, no satDNA was shared between *A. vittatum* and *Trogon* spp., consistent with their deeper divergence (21.4 My) [56]. These results support the “library hypothesis” [7], which proposes that related species share a set of satDNAs but differ in copy number, chromosomal distribution, and amplification dynamics. Similar cases of shared satDNAs despite long divergence times have also been documented in Corvidae and Paradisaeidae (~40 My) [9], in *Sula* spp. (~5.9 My) [27], and in *Turdus* spp. (~4.9 My) [25].

Regarding nucleotide composition, both Trogon species exhibited GC-rich satDNAs (mean 57.3% in *T. surrucura* and 58.4% in *T. melanurus*; Table 1 and Table 2), consistent with patterns in most birds [9,22,23,25,26,27]. In contrast, *A. vittatum* showed a predominance of AT-rich satDNAs (34.6% GC; Table 3), which is rare in birds but widespread in other groups such as insects [57,58], amphibians [59,60], fishes [61,62,63] and mammals [64,65,66]. The distinct GC composition and low similarity of satellitomes among Trogonidae genera are consistent with the “concerted evolution” model, whereby unequal crossing-over, gene conversion, and selection drive amplification or elimination of satellite repeats within genomes [4,5].

### 4.3. Chromosomal Distribution of TsuSatDNAs and TmeSatDNAs

The mapping of satDNAs to microchromosomes is common in birds, indicating that these small chromosomes harbor a high proportion of repetitive sequences [22,23,24,25,26,27]. A similar pattern occurs in trogons: in situ hybridization revealed a strong accumulation of satDNA in the pericentromeric/centromeric regions of microchromosomes in *T. surrucura* (Figure 4), and in silico mapping confirmed that macrochromosomes lack large satDNA clusters, carrying only a few sequences scattered across their length (Figure 2 and Figure 3).

Except for TsuSat07-200, all satDNAs localized to centromeric regions, consistent with the role of patterns described for other avian genomes [22,23,24,25,26,27]. Such centromeric enrichment is consistent with the essential role of satDNAs in ensuring chromosome stability during meiosis and mitosis [4,67,68]. Interestingly, chromosome-specific satDNAs were identified in *T. surrucura*: TsuSat05-399, TsuSat08-33, and TsuSat15-70 were exclusive to chromosome 2, while TsuSat11 and TsuSat13 localized exclusively to chromosome 1. TsuSat08-33 shared 69% similarity with TmeSat05-32, also restricted to chromosome 2 in *T. melanurus*. The distinct pattern in *T. surrucura* is the absence of TsuSat01-165 and TsuSat06-158 in chromosome 2, contrasting with other autosomes.

Comparative mapping using *G. gallus* and *Leucopternis albicollis* probes demonstrated that chromosome 2 in *T. surrucura* is homologous to the putative ancestral avian chromosome 3, whereas chromosome 1, homologous to the ancestral avian chromosome 1, underwent two pericentric inversions [33]. In both cases, the centromere remains intact, raising the question of why *Trogon* spp. harbor the chromosome pair 2 with exclusive satDNAs (TsuSat05-399, TsuSat08-33, TsuSat15-70) (Figure 4). One possible explanation is the rapid evolutionary rate of Trogonidae species [31]. In the evolutionary process, the chromosomal-specific satDNA may act as a postzygotic barrier throughout centromeric disruption on hybrids, as reported on *Drosophila* spp. hybrids [18,21]. Similar cases of chromosome-specific satDNAs have been reported in microchromosomes of *Turdus leucomelas* [25], *Sula* spp. [27], and *Jacana jacana* [23]. However, in these instances, the centromeric location could not be confirmed, as microchromosomes are punctiform and difficult to characterize morphologically. The in situ mapping on both *Trogon* spp. species and hybrids are necessary to confirm this hypothesis.

### 4.4. Trogon spp. Sex Chromosomes

In *T. surrucura*, the W chromosome, although similar in size to the Z chromosome [33], harbored only three satDNAs (TsuSat01-165, TsuSat02-31, TsuSat06-158), all restricted to centromeric regions and not exclusive to W (Figure 3). Additionally, no satDNA was in silico mapped on the W chromosome of both Trogon species (Figure 1 and Figure 2). Farias de Farias et al. [38] also reported the presence of the transposable element AviRTE at the W centromere. This finding contrasts with the common expectation that avian W chromosomes act as reservoirs of repetitive DNA [29], suggesting a distinct evolutionary dynamic in *Trogon*. Similar patterns of low repeat accumulation on W have been described in *Turdus* spp. [25]. Nonetheless, the presence of additional, undetected repetitive sequences on the W cannot be excluded. By contrast, W-specific satDNAs have been reported in other birds, such as *V. chilensis* (VchSat02) [22], *S. leucogaster* (SleSat04) [27], *Nannopterum brasilianum* (NbrSat07) [27], and *J. jacana* (JjaSat10) [23].

Notably, *T. surrucura* presented a Z-specific satDNA (TsuSat07-200) that strongly accumulated on its long arms (Figure 4). In silico mapping further identified three satDNAs located on the Z chromosome in both *Trogon* species (Figure 2 and Figure 3). Z-specific satDNAs are rare in birds but have been documented in *Turdus* spp., where TleSat10 is restricted to Z centromeres [25]. In most avian species studied by FISH, the Z chromosome either lacks satDNA signals (*S. leucogaster*, *N. brasilianum*, and *J. jacana*) or contains them at centromeres (*V. chilensis*, *Dromaius novaehollandiae*, and *Turdus* spp.) [22,23,24,25,27]. Moreover, the p arms of the Z chromosome in *T. surrucura* is largely composed of the transposable element AviRTE [33], reinforcing the idea that avian sex chromosomes, although not always major repositories of satDNA, may accumulate other types of repetitive sequences. Studies in Drosophila have shown that satDNAs located on homogametic sex chromosomes can contribute to reproductive isolation and speciation [17,18]. The accumulation of TsuSat07-200 on the Z chromosome of *T. surrucura*, and possibly in other taxa, indicates that satDNA-driven differentiation is not limited to haploid sex chromosomes.

## 5. Conclusions

In this study, we have provided the first comparative characterization of satellite DNAs in three trogonid species, *T. surrucura*, *T. melanurus*, and *A. vittatum*. Our results demonstrate that even closely related species can display highly divergent satellite landscapes. The two *Trogon* species followed the typical avian trend of GC-rich satDNA repeats and shared several satDNA families, whereas *A. vittatum* stood out as the first bird species described with predominantly AT-rich satDNAs and the lowest number of satDNA families reported to date in Aves. This striking reduction suggests lineage-specific repeat loss or reduced amplification in *A. vittatum*. Chromosomal mapping in *T*. *surrucura* further revealed unique features: chromosome-specific satDNAs restricted to pairs 1 and 2, which may enhance chromosomal stability and contribute to reproductive isolation, as well as a Z-specific satDNA strongly accumulated on the long arm of the chromosome. This last feature contrasts with the general avian pattern of Z-linked repeats restricted to centromeres and supports a potential role for sex-linked repeats in speciation.

Taken together, these findings demonstrate that satDNAs in Trogonidae exhibit both conserved and lineage-specific patterns of organization. They support the view that satDNAs function not only as structural elements of chromosomes but also as contributors to genome evolution, chromosomal diversification, and processes associated with reproductive isolation and speciation in birds. Moreover, the observed variation in satDNA distribution across lineages underscores their potential role as dynamic genomic elements influencing both stability and evolutionary changes.

## Figures and Tables

**Table 1:** Characteristics of *T. surrucura* DNA satellites.

SatDNA Family	Abundance	Divergence	A + T	G + C
TsuSat01-165	0.03326539	4.22	53.3	46.7
TsuSat02-31	0.02396841	18.98	58.1	41.9
TsuSat03-21	0.00984385	14.63	23.8	76.2
TsuSat04-31	0.00241935	11.06	38.7	61.3
TsuSat05-399	0.00228525	1406	55.6	44.4
TsuSat06-158	0.00123190	7.61	52.5	47.5
TsuSat07-200	0.00058407	9.55	55	45
TsuSat08-33	0.00055382	11.65	30.3	69.7
TsuSat09-52	0.00029597	11.18	32.7	67.3
TsuSat10-30	0.00020518	7.54	46.7	53.3
TsuSat11-16	0.00018562	5.47	37.5	62.5
TsuSat12-356	0.00016064	3.76	39.3	60.7
TsuSat13-31	0.00015431	9.67	35.5	64.5
TsuSat14-209	0.00014625	4.58	38.3	61.7
TsuSat15-70	0.00013374	19.80	50	50
TsuSat16-105	0.00008406	7.72	36.2	63.8
Total	0.07551780			
Mean			42.7	57.3

G + C = cytosine and guanine; A + T = adenine and thymine.

**Table 2:** Characteristics of *T. melanurus* DNA satellites.

SatDNA Family	Abundance	Divergence	A + T	G + C
TmeSat01-189	0.02480270	11.05	40.2	59.8
TmeSat02-165	0.01373952	9.82	50.3	49.7
TmeSat03-2458	0.00414056	6.50	50.6	49.4
TmeSat04-14	0.00243189	14.32	21.4	78.6
TmeSat05-32	0.00079474	13.60	28.1	71.9
TmeSat06-2022	0.00071064	3.64	33.5	66.5
TmeSat07-219	0.00042175	7.57	52.1	47.9
TmeSat08-178	0.00034514	10.96	47.2	52.8
TmeSat09-202	0.00032589	12.54	52	48
TmeSat10-93	0.00029909	19.27	58.1	41.9
TmeSat11-502	0.00027754	14.58	48	52
TmeSat12-356	0.00018984	3.73	38.8	61.2
TmeSat13-20	0.00016633	12.23	25	75
TmeSat14-209	0.00014953	8.91	38.3	61.7
TmeSat15-37	0.00012839	13.77	40.5	59.5
Total	0.04892355			
Mean			41.6	58.4

G + C = cytosine and guanine; A + T = adenine and thymine.

**Table 3:** Characteristics of *A. vittatum* DNA satellites.

SatDNA Family	Abundance	Divergence	A + T	C + G
AviSat01-166	0.01682297	8.92	59.6	40.4
AviSat02-24	0.00103356	9.50	79.2	20.8
AviSat03-334	0.00062750	14.14	57.4	42.6
Total	0.01848403			
Mean			65.4	34.6

G + C = cytosine and guanine; A + T = adenine and thymine.

**Table 4:** Similarity between satDNAs families of *T. surrucura* and *T. melanurus*.

Similarity	TsuSatDNA	TmeSatDNA
98%	TsuSat14-209	TmeSat14-209
92%	TsuSat12-356	TmeSat12-356
89%	TsuSat07-200	TmeSat09-202
85%	TsuSat03-21	TmeSat13-20
84%	TsuSat01-165	TmeSat02-165
77%	TsuSat02-31	TmeSat10-93
74%	TsuSat06-158	TmeSat02-165
71%	TsuSat08-33	TmeSat15-37
69%	TsuSat08-33	TmeSat05-32

**Table 5:** FISH signals of satellite DNAs of *T. surrucura*.

Satellite	FISH Signals
TsuSat01-165	centromere of macrochromosomes (except pair 2), microchromosomes, and centromere of W
TsuSat02-31	absent on one micro and the first seven pairs, centromere of the W chromosome
TsuSat03-21	38 microchromosomes
TsuSat04-31	20 microchromosomes
TsuSat05-399	centromere of pair 2
TsuSat06-158 *	strong on the centromere of pair 4 and weaker on other autosomes (except pair 2), weak on the centromere of W
TsuSat07-200	long arm of chromosome Z
TsuSat08-33	centromere of pair 2
TsuSat09-52	not amplified
TsuSat10-30	no signals
TsuSat11-16	centromere of pair 1
TsuSat12-356	not amplified
TsuSat13-31	centromere of pair 1
TsuSat14-209	no signals
TsuSat15-70	not amplified
TsuSat16-105	no signals

* The weak signals were not allowed to confirm the exact number of signals on microchromosomes.

## Data Availability

The sequencing reads have been deposited in the Sequence Read Archive (SRA-NCBI) and are available under the following accession number: *T. surrucura*-SRR35639635. The original contributions presented in this study are included in the article/Supplementary Material.

## Supplementary Materials

The following supporting information can be downloaded at https://www.mdpi.com/article/10.3390/genes16111301/s1. Figure S1: Landscape from *T. surrucura* (A), *T. melanurus* (B), and *A. vittatum* (C) DNA satellites in order of abundance; Table S1: Primers designed for the amplification of the DNA satellite of *T. surrucura* (TsuSat); Table S2: PCR protocol for the amplification of the DNA satellite of *T. surrucura* (TsuSat).

## References

[1] López-Flores I., Garrido-Ramos M.A. (2012). The Repetitive DNA Content of Eukaryotic Genomes. Genome Dyn..

[2] Yang H., Goubert C., Cotoras D.D., Dimitrov D., Graham N.R., Cerca J., Gillespie R.G. (2024). Consistent Accumulation of Transposable Elements in Species of the Hawaiian *Tetragnatha* Spiny-Leg Adaptive Radiation across the Archipelago Chronosequence. Evol. J. Linn. Soc..

[3] Ruiz-Ruano F.J., López-León M.D., Cabrero J., Camacho J.P.M. (2016). High-Throughput Analysis of the Satellitome Illuminates Satellite DNA Evolution. Sci. Rep..

[4] Thakur J., Packiaraj J., Henikoff S. (2021). Sequence, Chromatin and Evolution of Satellite DNA. Int. J. Mol. Sci..

[5] Garrido-Ramos M. (2017). Satellite DNA: An Evolving Topic. Genes.

[6] Mora P., Pita S., Montiel E.E., Rico-Porras J.M., Palomeque T., Panzera F., Lorite P. (2023). Making the Genome Huge: The Case of *Triatoma delpontei*, a Triatominae Species with More than 50% of Its Genome Full of Satellite DNA. Genes.

[7] Fry K., Salser W. (1977). Nucleotide Sequences of HS-α Satellite DNA from Kangaroo Rat *Dipodomys ordii* and Characterization of Similar Sequences in Other Rodents. Cell.

[8] Lisachov A., Rumyantsev A., Prokopov D., Ferguson-Smith M., Trifonov V. (2023). Conservation of Major Satellite DNAs in Snake Heterochromatin. Animals.

[9] Peona V., Kutschera V.E., Blom M.P.K., Irestedt M., Suh A. (2023). Satellite DNA Evolution in Corvoidea Inferred from Short and Long Reads. Mol. Ecol..

[10] Albuquerque L., Milani D., Martí E., Ferretti A.B.S.M., Rico-Porras J.M., Mora P., Lorite P., Ziabari O.S., Brisson J.A., Palacios-Gimenez O.M. (2025). Exploring the Satellitome of the Pest Aphid *Acyrthosiphon pisum* (Hemiptera, Aphididae): Insights Into Genome Organization and Intraspecies Evolution. Genome Biol. Evol..

[11] Coen E., Strachan T., Dover G. (1982). Dynamics of Concerted Evolution of Ribosomal DNA and Histone Gene Families in the Melanogaster Species Subgroup of *Drosophila*. J. Mol. Biol..

[12] Dover G. (1982). Molecular Drive: A Cohesive Mode of Species Evolution. Nature.

[13] Camacho J.P.M., Cabrero J., López-León M.D., Martín-Peciña M., Perfectti F., Garrido-Ramos M.A., Ruiz-Ruano F.J. (2022). Satellitome Comparison of Two Oedipodine Grasshoppers Highlights the Contingent Nature of Satellite DNA Evolution. BMC Biol..

[14] Lisachova L., Lisachov A., Romanenko S., Davletshina G., Altmanová M., Rovatsos M., Kratochvíl L., Giovannotti M., Nazarov R., Okshtein I. (2025). Concerted Evolution of Genus-Specific Centromeric Satellite DNA in *Eremias* (Lacertidae, Reptilia). Cytogenet. Genome Res..

[15] Feliciello I., Picariello O., Chinali G. (2006). Intra-Specific Variability and Unusual Organization of the Repetitive Units in a Satellite DNA from Rana Dalmatina: Molecular Evidence of a New Mechanism of DNA Repair Acting on Satellite DNA. Gene.

[16] Feliciello I., Picariello O., Chinali G. (2005). The First Characterisation of the Overall Variability of Repetitive Units in a Species Reveals Unexpected Features of Satellite DNA. Gene.

[17] Ferree P.M., Barbash D.A. (2009). Species-Specific Heterochromatin Prevents Mitotic Chromosome Segregation to Cause Hybrid Lethality in *Drosophila*. PLoS Biol..

[18] Cattani M.V., Presgraves D.C. (2012). Incompatibility between X Chromosome Factor and Pericentric Heterochromatic Region Causes Lethality in Hybrids between *Drosophila melanogaster* and Its Sibling Species. Genetics.

[19] Puppo I.L., Saifitdinova A.F., Tonyan Z.N. (2020). The Role of Satellite DNA in Causing Structural Rearrangements in Human Karyotype. Russ. J. Genet..

[20] Shatskikh A.S., Kotov A.A., Adashev V.E., Bazylev S.S., Olenina L.V. (2020). Functional Significance of Satellite DNAs: Insights From *Drosophila*. Front. Cell Dev. Biol..

[21] Jagannathan M., Yamashita Y.M. (2021). Defective Satellite DNA Clustering into Chromocenters Underlies Hybrid Incompatibility in *Drosophila*. Mol. Biol. Evol..

[22] Kretschmer R., Toma G.A., Deon G.A., Santos N.D., Dos Santos R.Z., Utsunomia R., Porto-Foresti F., Gunski R.J., Garnero A.D.V., Liehr T. (2024). Satellitome Analysis in the Southern Lapwing (*Vanellus chilensis*) Genome: Implications for SatDNA Evolution in Charadriiform Birds. Genes.

[23] De Oliveira A.M., Souza G.M., Toma G.A., Dos Santos N., Dos Santos R.Z., Goes C.A.G., Deon G.A., Setti P.G., Porto-Foresti F., Utsunomia R. (2024). Satellite DNAs, Heterochromatin, and Sex Chromosomes of the Wattled Jacana (Charadriiformes; Jacanidae): A Species with Highly Rearranged Karyotype. Genome.

[24] Setti P.G., Deon G.A., Zeni Dos Santos R., Goes C.A.G., Garnero A.D.V., Gunski R.J., De Oliveira E.H.C., Porto-Foresti F., De Freitas T.R.O., Silva F.A.O. (2024). Evolution of Bird Sex Chromosomes: A Cytogenomic Approach in Palaeognathae Species. BMC Ecol. Evo..

[25] Souza G.M., Kretschmer R., Toma G.A., De Oliveira A.M., Deon G.A., Setti P.G., Zeni Dos Santos R., Goes C.A.G., Del Valle Garnero A., Gunski R.J. (2024). Satellitome Analysis on the Pale-Breasted Thrush *Turdus leucomelas* (Passeriformes; Turdidae) Uncovers the Putative Co-Evolution of Sex Chromosomes and Satellite DNAs. Sci. Rep..

[26] Souza G.M., Vidal J.A.D., Utsunomia R., Deon G.A., De Oliveira E.H.C., Franca R.T., Porto-Foresti F., Liehr T., De Souza F.H.S., Kretschmer R. (2025). Cytogenomic Analysis in Seriemas (Cariamidae): Insights into an Atypical Avian Karyotype. J. Hered..

[27] Pozzobon L.C., Dos Santos N., Utsunomia R., Porto-Foresti F., Cioffi M.D.B., Kretschmer R., De Freitas T.R.O. (2025). Satellite DNA Mapping in Suliformes (Aves): Insights into the Evolution of the Multiple Sex Chromosome System in *Sula* Spp.. Genes.

[28] Huang Z., Xu Z., Bai H., Huang Y., Kang N., Ding X., Liu J., Luo H., Yang C., Chen W. (2023). Evolutionary Analysis of a Complete Chicken Genome. Proc. Natl. Acad. Sci. USA.

[29] Peona V., Palacios-Gimenez O.M., Blommaert J., Liu J., Haryoko T., Jønsson K.A., Irestedt M., Zhou Q., Jern P., Suh A. (2021). The Avian W Chromosome Is a Refugium for Endogenous Retroviruses with Likely Effects on Female-Biased Mutational Load and Genetic Incompatibilities. Phil. Trans. R. Soc. B.

[30] Winkler D.W., Billerman S.M., Lovette I.J., Billerman S.M., Keeney B.K., Rodewald P.G., Schulenberg T.S. (2020). Trogons (Trogonidae). Birds of the World.

[31] Oliveros C.H., Andersen M.J., Hosner P.A., Mauck W.M., Sheldon F.H., Cracraft J., Moyle R.G. (2020). Rapid Laurasian Diversification of a Pantropical Bird Family during the Oligocene–Miocene Transition. Ibis.

[32] Bian X., Cai H., Ning S., Xong X., Han L., Chen X., Li Q., Zhang H., Liu A., Yang L. (1991). Studies on the Karyotypes of Birds XII. 15 Species of Nonpasserines. (Aves). Zool. Res..

[33] Degrandi T.M., Del Valle Garnero A., O’Brien P.C.M., Ferguson-Smith M.A., Kretschmer R., De Oliveira E.H.C., Gunski R.J. (2017). Chromosome Painting in *Trogon s. surrucura* (Aves, Trogoniformes) Reveals a Karyotype Derived by Chromosomal Fissions, Fusions, and Inversions. Cytogenet. Genome Res..

[34] Kretschmer R., Ferguson-Smith M., De Oliveira E. (2018). Karyotype Evolution in Birds: From Conventional Staining to Chromosome Painting. Genes.

[35] O’Connor R.E., Kretschmer R., Romanov M.N., Griffin D.K. (2024). A Bird’s-Eye View of Chromosomic Evolution in the Class Aves. Cells.

[36] Griffin D.K., Kretschmer R., Larkin D.M., Srikulnath K., Singchat W., Narushin V.G., O’Connor R.E., Romanov M.N. (2025). Avian Cytogenomics: Small Chromosomes, Long Evolutionary History. Genes.

[37] Degrandi T.M., Barcellos S.A., Costa A.L., Garnero A.D.V., Hass I., Gunski R.J. (2020). Introducing the Bird Chromosome Database: An Overview of Cytogenetic Studies in Birds. Cytogenet. Genome Res..

[38] Farias De Farias N., Gunski R.J., Del Valle Garnero A., Cañedo A.D., Herculano Correa De Oliveira E., Oliveira Silva F.A., Torres F.P. (2024). Chromosome Mapping of Retrotransposon AviRTE in a Neotropical Bird Species: *Trogon surrucura* (Trogoniformes; Trogonidae). Genome.

[39] Kretschmer R., De Souza M.S., Furo I.D.O., Romanov M.N., Gunski R.J., Garnero A.D.V., De Freitas T.R.O., De Oliveira E.H.C., O’Connor R.E., Griffin D.K. (2021). Interspecies Chromosome Mapping in Caprimulgiformes, Piciformes, Suliformes, and Trogoniformes (Aves): Cytogenomic Insight into Microchromosome Organization and Karyotype Evolution in Birds. Cells.

[40] De Oliveira Furo I., Kretschmer R., Dos Santos M.S., De Lima Carvalho C.A., Gunski R.J., O’Brien P.C.M., Ferguson-Smith M.A., Cioffi M.B., De Oliveira E.H.C. (2017). Chromosomal Mapping of Repetitive DNAs in *Myiopsitta monachus* and *Amazona aestiva* (Psittaciformes, Psittacidae) with Emphasis on the Sex Chromosomes. Cytogenet. Genome Res..

[41] Flynn J.M., Hubley R., Goubert C., Rosen J., Clark A.G., Feschotte C., Smit A.F. (2020). RepeatModeler2 for Automated Genomic Discovery of Transposable Element Families. Proc. Natl. Acad. Sci. USA.

[42] Smit A., Hubley R., Green P. RepeatMasker Open-5.0 2013–2015. http://www.repeatmasker.org.

[43] Novák P., Ávila Robledillo L., Koblížková A., Vrbová I., Neumann P., Macas J. (2017). TAREAN: A Computational Tool for Identification and Characterization of Satellite DNA from Unassembled Short Reads. Nucleic Acids Res..

[44] Bolger A.M., Lohse M., Usadel B. (2014). Trimmomatic: A Flexible Trimmer for Illumina Sequence Data. Bioinformatics.

[45] Schmieder R., Edwards R. (2011). Fast Identification and Removal of Sequence Contamination from Genomic and Metagenomic Datasets. PLoS ONE.

[46] Edgar R.C. (2004). MUSCLE: Multiple Sequence Alignment with High Accuracy and High Throughput. Nucleic Acids Res..

[47] Rico-Porras J.M., Mora P., Palomeque T., Montiel E.E., Cabral-de-Mello D.C., Lorite P. (2024). Heterochromatin Is Not the Only Place for satDNAs: The High Diversity of satDNAs in the Euchromatin of the Beetle *Chrysolina americana* (Coleoptera, Chrysomelidae). Genes.

[48] Kretschmer R., dos Santos M.d.S., Furo I.D.O., De Oliveira E.H.C., Cioffi M.D.B., Liehr T. (2022). FISH—In Birds. Cytogenetics and Molecular Cytogenetics.

[49] Sumner A.T. (1972). A Simple Technique for Demonstrating Centromeric Heterochromatin. Exp. Cell Res..

[50] Kapusta A., Suh A., Feschotte C. (2017). Dynamics of Genome Size Evolution in Birds and Mammals. Proc. Natl. Acad. Sci. USA.

[51] Card D.C., Jennings W.B., Edwards S.V. (2023). Genome Evolution and the Future of Phylogenomics of Non-Avian Reptiles. Animals.

[52] Wang Z.-J., Chen G.-J., Zhang G.-J., Zhou Q. (2021). Dynamic Evolution of Transposable Elements, Demographic History, and Gene Content of Paleognathous Birds. Zool. Res..

[53] Li B.-P., Kang N., Xu Z.-X., Luo H.-R., Fan S.-Y., Ao X.-H., Li X., Han Y.-P., Ou X.-B., Xu L.-H. (2025). Transposable Elements Shape the Landscape of Heterozygous Structural Variation in a Bird Genome. Zool. Res..

[54] Ricci M., Peona V., Guichard E., Taccioli C., Boattini A. (2018). Transposable Elements Activity Is Positively Related to Rate of Speciation in Mammals. J. Mol. Evol..

[55] Galbraith J.D., Kortschak R.D., Suh A., Adelson D.L. (2021). Genome Stability Is in the Eye of the Beholder: CR1 Retrotransposon Activity Varies Significantly across Avian Diversity. Genome Biol. Evol..

[56] Kumar S., Stecher G., Suleski M., Hedges S.B. (2017). TimeTree: A Resource for Timelines, Timetrees, and Divergence Times. Mol. Biol. Evol..

[57] De Lima L.G., Ruiz-Ruano F.J. (2022). In-Depth Satellitome Analyses of 37 *Drosophila* Species Illuminate Repetitive DNA Evolution in the *Drosophila* Genus. Genome Biol. Evol..

[58] Pereira J.A., Cabral-de-Mello D.C., Lopes D.M. (2023). The Satellite DNAs Populating the Genome of *Trigona hyalinata* and the Sharing of a Highly Abundant satDNA in *Trigona* Genus. Genes.

[59] Silva M.J.d., Gazoni T., Haddad C.F.B., Parise-Maltempi P.P. (2023). Analysis in *Proceratophrys boiei* Genome Illuminates the Satellite DNA Content in a Frog from the Brazilian Atlantic Forest. Front. Genet..

[60] Souza L.H.B., Ferro J.M., Milanez H.M., Haddad C.F.B., Lourenço L.B. (2025). New Insights into the Sex Chromosome Evolution of the Common Barker Frog Species Complex (Anura, Leptodactylidae) Inferred from Its Satellite DNA Content. Biomolecules.

[61] Boštjančić L.L., Bonassin L., Anušić L., Lovrenčić L., Besendorfer V., Maguire I., Grandjean F., Austin C.M., Greve C., Hamadou A.B. (2021). The *Pontastacus leptodactylus* (Astacidae) Repeatome Provides Insight Into Genome Evolution and Reveals Remarkable Diversity of Satellite DNA. Front. Genet..

[62] Goes C.A.G., Dos Santos R.Z., Aguiar W.R.C., Alves D.C.V., Silva D.M.Z.D.A., Foresti F., Oliveira C., Utsunomia R., Porto-Foresti F. (2022). Revealing the Satellite DNA History in *Psalidodon* and *Astyanax* Characid Fish by Comparative Satellitomics. Front. Genet..

[63] Goes C.A.G., Dos Santos N., Rodrigues P.H.D.M., Stornioli J.H.F., Silva A.B.D., Dos Santos R.Z., Vidal J.A.D., Silva D.M.Z.D.A., Artoni R.F., Foresti F. (2022). The Satellite DNA Catalogues of Two Serrasalmidae (Teleostei, Characiformes): Conservation of General satDNA Features over 30 Million Years. Genes.

[64] Dias C.A.R., Kuhn G.C.S., Svartman M., Santos Júnior J.E.D., Santos F.R., Pinto C.M., Perini F.A. (2021). Identification and Characterization of Repetitive DNA in the Genus *Didelphis* Linnaeus, 1758 (Didelphimorphia, Didelphidae) and the Use of Satellite DNAs as Phylogenetic Markers. Genet. Mol. Biol..

[65] Gutiérrez J., Aleix-Mata G., Montiel E.E., Cabral-de-Mello D.C., Marchal J.A., Sánchez A. (2022). Satellitome Analysis on *Talpa aquitania* Genome and Inferences about the satDNAs Evolution on Some Talpidae. Genes.

[66] Aleix-Mata G., Montiel E.E., Mora P., Yurchenko A., Rico-Porras J.M., Anguita F., Palomo F., Marchal J.A., Rovatsos M., Sánchez A. (2025). Satellitome Analysis on *Microtus thomasi* (Arvicolinae) Genome, a Mammal Species with High Karyotype and Sex Chromosome Variations. Genome.

[67] Melters D.P., Bradnam K.R., Young H.A., Telis N., May M.R., Ruby J., Sebra R., Peluso P., Eid J., Rank D. (2013). Comparative Analysis of Tandem Repeats from Hundreds of Species Reveals Unique Insights into Centromere Evolution. Genome Biol..

[68] Talbert P.B., Henikoff S. (2020). What Makes a Centromere?. Exp. Cell Res..

