# Supplementary material for: Contrasting Satellitomes in New World and African Trogons (Aves, Trogoniformes)
Source: Genes (Basel). 2025 Nov 1;16(11):1301. doi: 10.3390/genes16111301 (PMC12651973; doi:10.3390/genes16111301)

GCAT TACG GCAT genes

MDPI

Article

Contrasting Satellitomes in New World and African Trogons (Aves, Trogoniformes)

Luciano Cesar Pozzobon [1], Jhon Alex Dziechciarz Vidal [2], Felipe Lagreca Bitencour [1], Analía Del Valle Garnero [3], Ricardo José Gunski [3], Hélio Gomes da Silva Filho [4], Fabio Porto-Foresti [4], Ricardo Utsunomia [4], Marcelo de Bello Cioffi [2], Thales Renato Ochotorena de Freitas [1] and Rafael Kretschmer [1,*]

[1] Laboratório de Citogenética e Evolução, Departamento de Genética, Instituto de Biociências, Universidade Federal do Rio Grande do Sul, Porto Alegre 91509-900, Brazil; lcpozzobon48@gmail.com (L.C.P.); felipe.lagreca@ufrgs.br (F.L.B.); thales.freitas@ufrgs.br (T.R.O.d.F.)

[2] Laboratório de Citogenética Evolutiva, Departamento de Genética e Evolução, Universidade Federal de São Carlos, São Carlos 13565-905, Brazil; jhonalex279@gmail.com (J.A.D.V.); mbcioffi@ufscar.br (M.d.B.C.)

[3] Laboratório de Diversidade Genética Animal, Universidade Federal do Pampa, São Gabriel 97300-000, Brazil; analiagarnero@unipampa.edu.br (A.D.V.G.); ricardogunski@unipampa.edu.br (R.J.G.)

[4] Faculdade de Ciências, Universidade Estadual Paulista, Bauru 13506-900, Brazil; hg.silva@unesp.br (H.G.d.S.F.); fp.foresti@unesp.br (F.P.-F.); ricardo.utsunomia@unesp.br (R.U.)

* Correspondence: rafael.kretschmer@ufrgs.br

Supplementary material

Supplementary Table S1. Primers designed for amplification of DNA satellite of *T. surrucura* (TsuSat)

TsuSat	Foward	Reverse
TsuSat01-165	CTCAAGCTCCAGACAAGCT	TTGTGAGCAGTGCAGGAGA
TsuSat02-31	Biotinylated Oligonucleotide	
TsuSat03-21	Biotinylated Oligonucleotide	
TsuSat04-31	Biotinylated Oligonucleotide	
TsuSat05-399	TAGGAAGGAAAACCAGTGTT	ACACAATCAGTCTGGGAG
TsuSat06-158	AGGCTGCCAAATCCGAG	CAGCAGCCCTCTCTTTA
TsuSat07-200	GATTACCCTTGTCCAGGT	GTGGGGATATAGGGTGA
TsuSat08-33	Biotinylated Oligonucleotide	
TsuSat09-52	CCCTCTGGCTCCCCA	AGGAACTGGGAGCACTG
TsuSat10-30	Biotinylated Oligonucleotide	
TsuSat11-16	Biotinylated Oligonucleotide	
TsuSat12-356	AACCGCAGCAAGCGGG	GGTAGCTTGAGGGTGCG
TsuSat13-31	Biotinylated Oligonucleotide	
TsuSat14-209	GGCGAGATGGTGGCAC	ATGTCCCTACCCAGGCA
TsuSat15-70	CCAAAGAAGGCCAAAGAG	CCCTCTTTGACCTTCTTCA
TsuSat16-105	GCTCCGGGGTGGTAACAA	CCTGACGGCCTGTTACTA

 https://doi.org/10.3390/genes16111301

Supplementary Table S2. PCR protocol for amplification of the DNA satellite of *T. surrucura* (TsuSat)

Satellite	Desnaturation	Cycles	Desnaturation	Annealing	Amplification	Final amplification	Concentration (ng/ul)
TsuSat01-165	95 °C for 5 min	35	95 °C for 45 s	51 °C for 35 s	72 °C for 1 min	72 °C for 10 min	0.01
TsuSat02-31	Biotinylated Oligonucleotide						
TsuSat03-21	Biotinylated Oligonucleotide						
TsuSat04-31	Biotinylated Oligonucleotide						
TsuSat05-399	95 °C for 5 min	35	95 °C for 45 s	52 °C for 35 seg	72 °C for 1 min	72 °C for 10 min	0.1
TsuSat06-158	95 °C for 5 min	35	95 °C for 45 s	52 °C for 35 s	72 °C for 1 min	72 °C for 10 min	0.01
TsuSat07-200	95 °C for 5 min	35	95 °C for 45 s	54 °C for 35 s	72 °C for 1 min	72 °C for 10 min	0.1
TsuSat08-33	Biotinylated Oligonucleotide						
TsuSat09-52	fail to amplify						
TsuSat10-30	Biotinylated Oligonucleotide						
TsuSat11-16	Biotinylated Oligonucleotide						
TsuSat12-356	fail to amplify						
TsuSat13-31	Biotinylated Oligonucleotide						
TsuSat14-209	95 °C for 5 min	35	95 °C for 45 s	55 °C for 35 s	72 °C for 1 min	72 °C for 10 min	0.01
TsuSat15-70	fail to amplify						
TsuSat16-105	95 °C for 5 min	35	95 °C for 45 s	58 °C for 35 s	72 °C for 1 min	72 °C for 10 min	0.1

Supplementary Figure S1. Landscape from *T. surrucura* (A), *T. melanurus* (B) and *A. vittatum* (C) DNA satellites in order of abundance

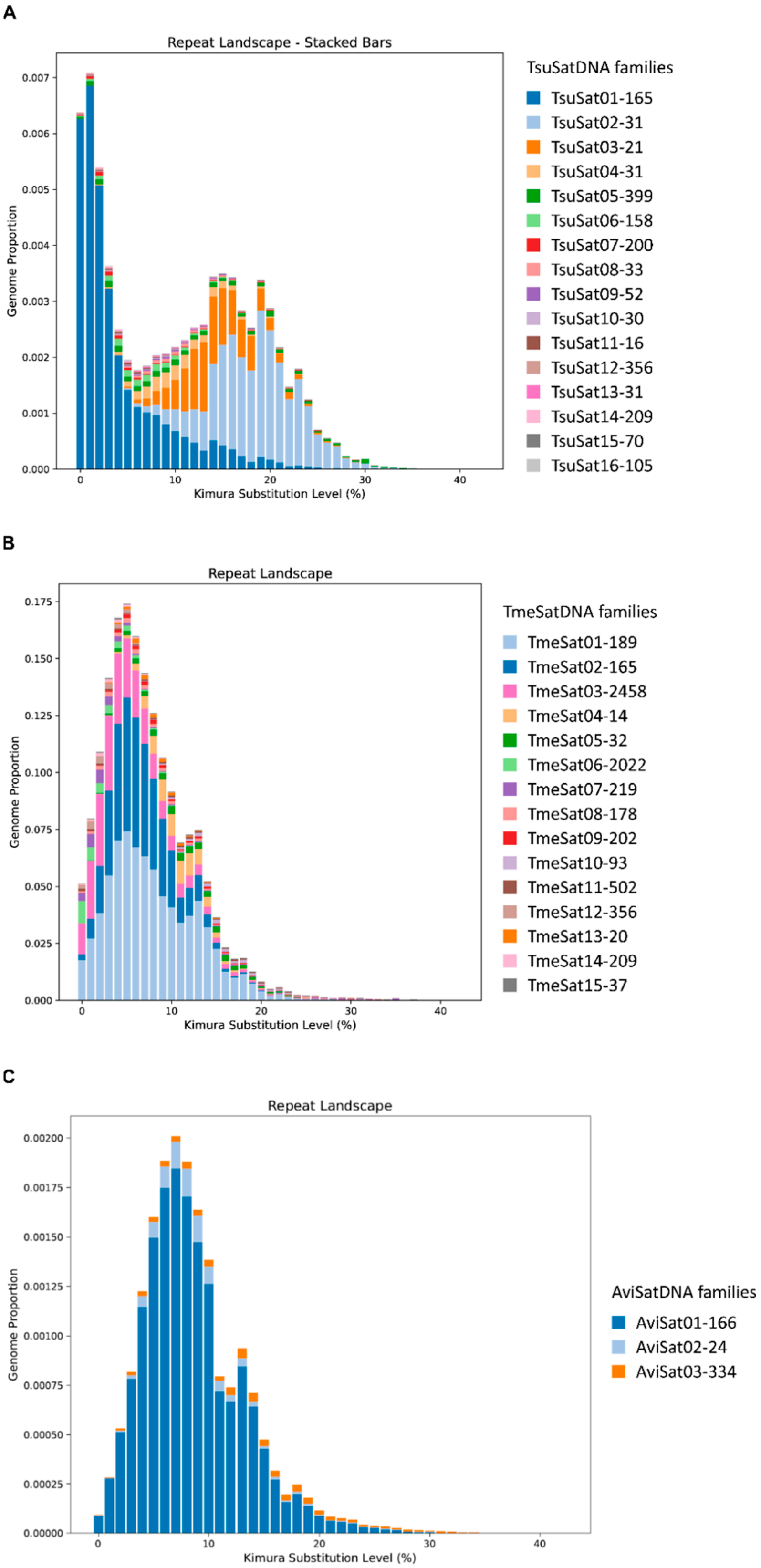

Supplement: Supplementary file 1 [file genes-16-01301-s001.zip › genes-3931777-supplementary.pdf]
